# Case Report: Pleural effusion in Wilms tumor – always malignant?

**DOI:** 10.12688/f1000research.138794.1

**Published:** 2023-08-30

**Authors:** Keta Vagha, Patel Zeeshan Jameel, Jayant Vagha, Ashish R. Varma, Rupesh Rao

**Affiliations:** 1 Pediatrics, Jawaharlal Nehru Medical College, Wardha, Maharashtra, 442001, India; 2 Hemato-Oncology and Bone Marrow Transplant, Royal Manchester Children's Hospital, Manchester, M139WL, UK

**Keywords:** Wilms tumor, pleural effusion, pulmonary metastasis

## Abstract

Wilms tumor (WT) is the most common renal malignancy seen in pediatric patients. Although lungs are the most common site of metastasis in Wilms tumor, non-malignant pleural effusion has been infrequently reported. Here, we report a case of an eleven-year-old female who presented with an abdominal mass and progressive breathlessness. On further evaluation, she was found to have a right-sided Wilms tumor with ipsilateral massive pleural effusion. The effusion resolved almost completely after four weeks of chemotherapy. We conclude that patients suffering from Wilms tumor presenting with pleural effusion need not be synonymous with metastatic disease and can have a favorable prognosis.

**Abbreviations**
CT: Computed tomography
CECT: Contrast enhanced computed tomography
IVC: Inferior vena cava
SIOP: International Society of Pediatric Oncology
vWF: von Willebrand factor
WT: Wilms tumor

## Introduction

Wilms tumors are responsible for approximately 6% of all malignancies and more than 95% of renal malignancies in the pediatric age group. ^1^ Early diagnosis, risk stratification, stage-based management and improved neo-adjuvant therapies have greatly improved the overall five-year survival up to >90%. ^2^ Wilms tumor is most often diagnosed clinically as an incidental discovery of an asymptomatic abdominal mass by parents or attending pediatrician. Other common symptoms include abdominal pain, gross painless hematuria, constitutional symptoms, and hypertension. Rarely, fatal pulmonary embolism, hematological abnormalities and pleural effusion have been reported in children with Wilms tumor. ^3^ Common sites for metastasis in advanced cases include abdominal lymph nodes, lungs and less often, liver and bone. Here, we report a rare case of Wilms tumor presenting clinically with a massive pleural effusion.

## Case presentation

### Patient information

An eleven-year-old, previously healthy adolescent girl from central India presented with a one-week history of abdominal distension with abdominal pain and a five-day history of progressive breathlessness.

### Clinical findings

Upon initial physical examination, she had tachycardia (heart rate [HR]-130/min), tachypnea (respiratory rate [RR]-40/min), a normal blood pressure (110/80 mmHg) and was maintaining SpO _2_ on room air. Respiratory system examination showed tracheal deviation to the left, stony dull percussive note and absent breath sounds on the right side suggestive of right-sided pleural effusion. Examination of the abdomen showed a well-defined, firm, mildly tender mass (12×14 cm) palpable in right lumbar, hypochondrium, epigastric and umbilical regions. The upper border of the mass was distinctly palpable from the liver. There were no associated congenital malformations.

### Diagnostic assessment

**Figure 1.**
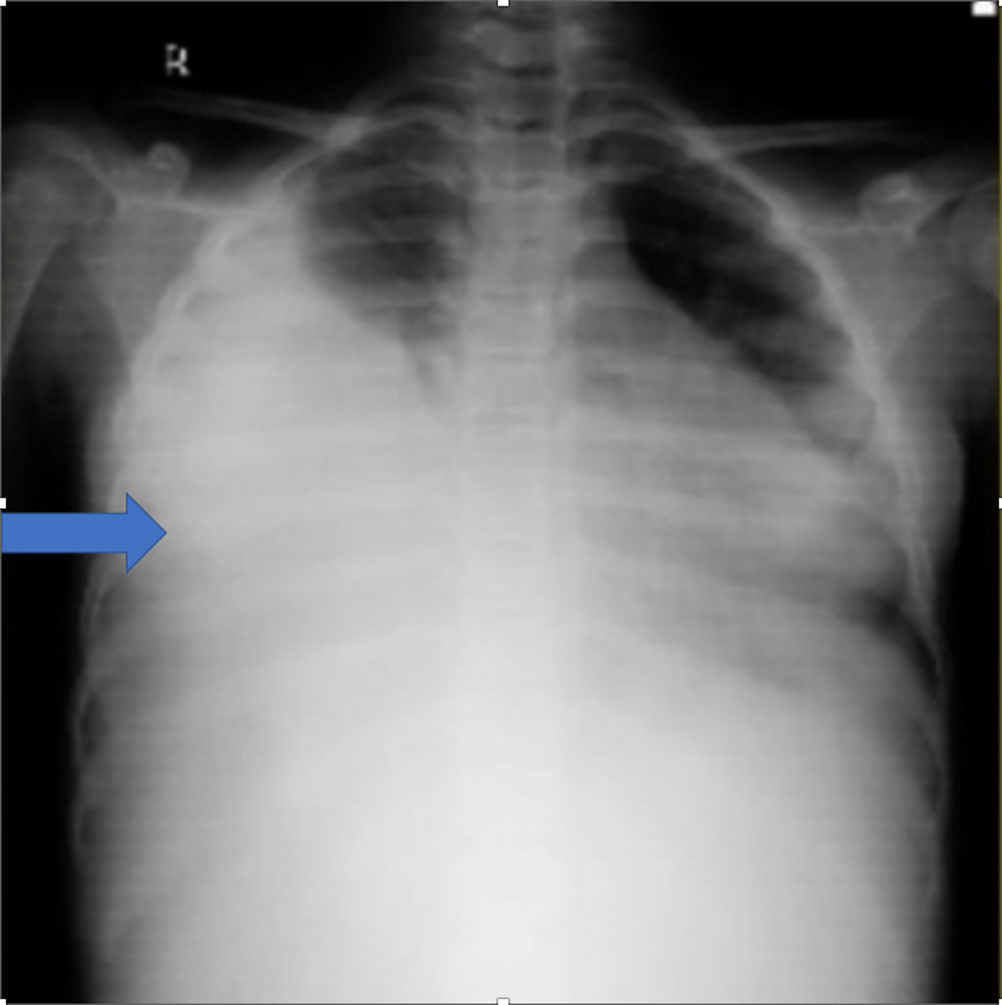
Chest X-ray showing right sided massive pleural effusion with mediastinal shift towards left.

Her hematological parameters were within limits, except thrombocytosis. Serum biochemistry was normal. Liver and kidney function tests were within limits. Urine analysis was also normal. Therapeutic thoracocentesis was done and around 750 mL of pleural fluid was aspirated gradually over the course of 48 hours, following which she improved symptomatically. Pleural fluid analysis revealed a blood-stained, sterile fluid, with protein content of 4.4 gm/dL, glucose of 91 mg/dL, and LDH of 1043 IU/L. Fluid cytology revealed markedly increased lymphoid cell with plenty of red blood cells. No malignant cells were visualized. Chest radiograph was suggestive of a massive right sided pleural effusion ( Figure 1).

**Figure 2.**
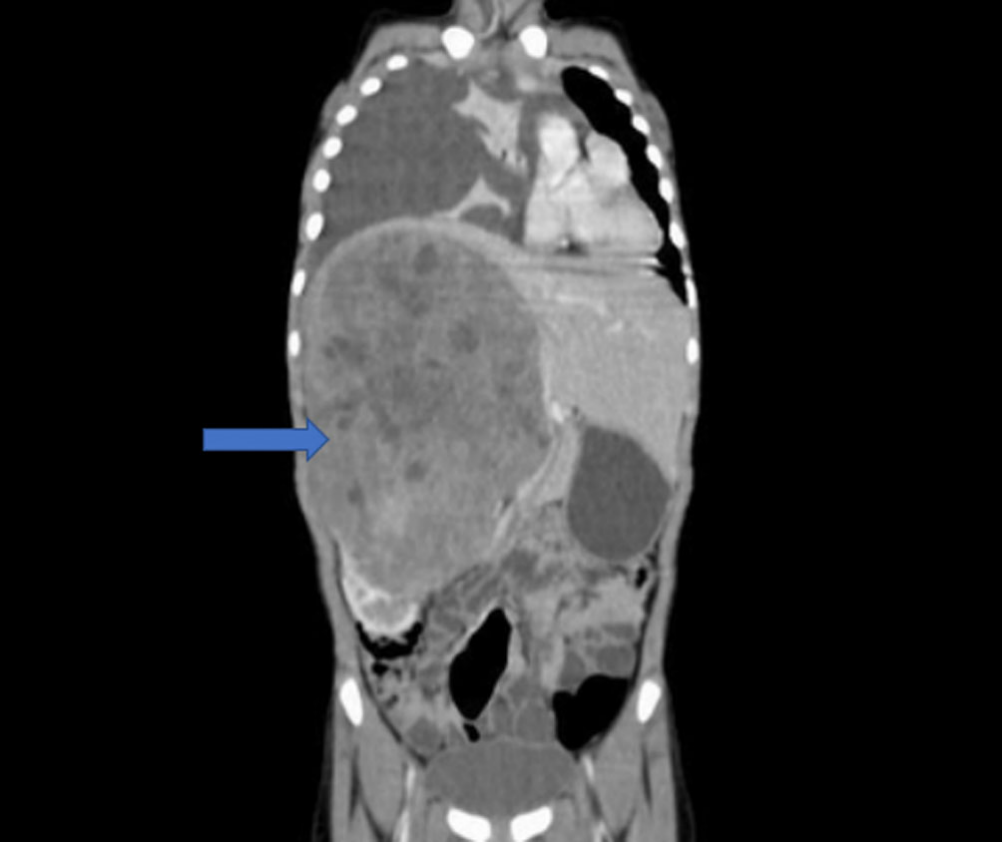
CECT abdomen showing a large heterogeneously enhancing mass with necrotic areas in the right retroperitoneum having a “claw shape” arising from the right kidney and extending across the midline to the left side with a normal left kidney. A right-sided massive pleural effusion with adjacent passive atelectasis can be seen.

Contrast enhanced computed tomography (CECT) of abdomen showed a large, heterogeneously enhancing mass (22×16×14 cm) with multiple necrotic areas arising from the mid and upper pole of the right kidney ( Figure 2).

### Diagnosis

On the basis of the above findings, a diagnosis of Wilms tumor with right-sided pleural effusion was made.

### Therapeutic intervention

**Figure 3.**
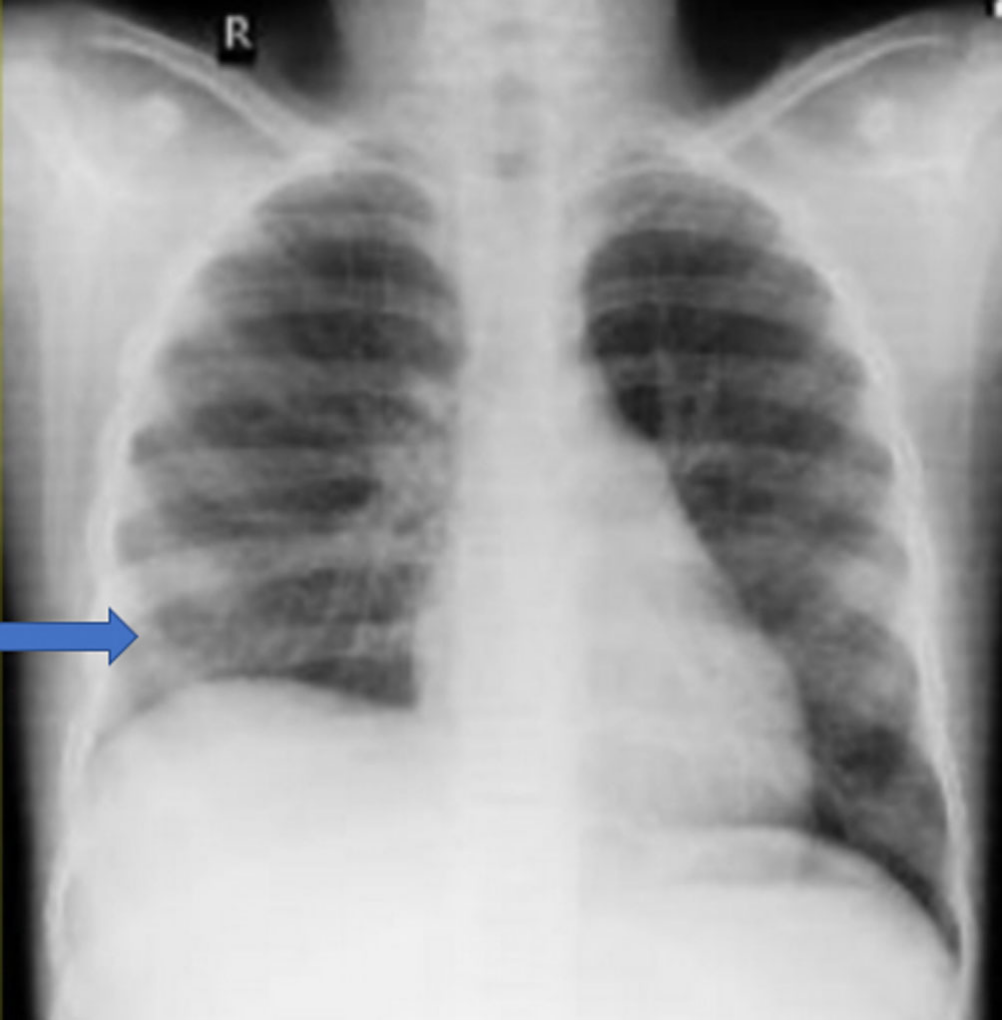
Chest X-ray showing complete resolution of pleural effusion after four weeks.

As per the International Society of Pediatric Oncology (SIOP), her management plan included preoperative chemotherapy followed by radical nephrectomy and post-operative chemotherapy. She received six cycles of chemotherapy prior to surgery comprising of vincristine (1.5 mg/m ^2^), actinomycin D (45 mcg/kg) and adriamycin (50 mg/m ^2^). Her pleural effusion completely resolved after four weeks of chemotherapy without the need for further thoracocentesis ( Figure 3). She then underwent right radical nephrectomy. However, during surgery the mass was found to be densely adherent to the inferior vena cava (IVC) across its length as well as to posterior aspect of liver and diaphragm. Some residual mass adherent to IVC was left behind. Histopathological examination of the specimen was suggestive of Wilms tumor (SIOP stage III) with no lymph nodal metastasis. In view of the residual disease, she received post-operative radiotherapy with a total dose of 10.8 grays to the abdomen.

### Follow up and outcome of the intervention

Further, as planned, she was started on weekly chemotherapy with vincristine, actinomycin D and adriamycin for 24 cycles. At time of writing, she has completed all her cycles with no further complications. Further management plan includes surveillance ultrasonography for abdominal recurrence or development of a second primary tumor in the contralateral kidney and chest CT for pulmonary metastasis after three months.

### Informed consent

Informed written consent was obtained from the patient and her parents for publication of this case.

## Discussion

The current case is interesting because of the unusual clinical presentation of pleural effusion. Since pleural effusion shows the involvement of an organ system distant from the primary tumor site, there is a tendency to think of metastatic disease in such cases. However, there were no signs of primary pulmonary metastasis in this case. Therefore, the present case highlights that those patients suffering from Wilms tumor presenting with pleural effusion need not be synonymous with metastatic disease and can have a favorable prognosis.

The most frequent site for metastasis in Wilms tumor is the lung, occurring in up to >90% of patients with metastatic disease. Rarely, pleural metastasis has also been documented. Pleural effusion is a rarely presenting feature in children with Wilms tumor. The incidence of pleural effusion has been reported to be 4.3%. ^4^ Different mechanisms implicated in the causation of pleural effusion are pleural metastasis, hypoproteinemia secondary to either chemotherapy or radiation-induced transient liver injury, or unrelated causes such as chylous exudate due to post-surgical lymphatic damage with associated infection. ^5^ Sympathetic effusion due to proximity of tumor to diaphragm or damage to diaphragm due to adhesion may be the cause in our case. Even though pleural effusion is seen in patients with Wilms tumor, massive effusions are rarely seen so as to cause respiratory distress as in our case. We were able to find at least six publications (17 children) of Wilms tumor with pleural effusion with pulmonary metastasis being reported in four children which are summarised in Table 1. ^4^^–^^9^ The significance of pleural effusion in these groups of patients is the fact that it dramatically upgrades the staging of tumor and therefore, changes the management of the patient. In a study by Wong *et al.,* ^10^ the malignant positivity of pleural effusion in WT with pleural effusion was found to be 35%. In stark contrast, a retrospective analysis done at St. Jude Children’s Research Hospital, Memphis, Tennessee, USA over 16-year period detected that there were no signs of metastasis in children with WT presenting with pleural effusion. ^4^ The treatment modality for WT with pulmonary metastasis includes chemotherapy with vincristine, actinomycin D and Adriamycin along with lung radiation therapy. The inappropriate upstaging of WT leads to over-treatment with consequent treatment-related toxicities. Pulmonary fibrosis and diffuse interstitial pneumonitis are complications secondary to lung radiation therapy for metastatic WT. Dilated cardiomyopathy is a potentially life-threatening complication due to Adriamycin by virtue of its ability to cause myocardial injury; it may also act as a radiosensitiser which further increases the potential for myocardial damage leading to reduced overall survival. Hence, appropriate staging as well as management are of utmost importance.

**Table 1.** Summary of studies reporting pleural effusion in Wilms tumor.

S.No	Author (Year of publication)	Patient demographics (Age in years; sex)	Pleural fluid analysis	Implicated etiology	Treatment given
1	Presented case	11 years; Female	Non-malignant effusion	Unknown	Therapeutic thoracocentesis followed by chemotherapy
2	Corey *et al.* (2004) ^4^	10 Children	Non-malignant effusion in all cases	Unknown	Chemotherapy alone in 8 cases; Additional pulmonary irradiation in 2 cases (Stage IV)
3	Betkerur and Lanzkowsky (1977) ^5^	Case 1: 4 year 8 months; Male	Malignant effusion	Secondary to pleural metastasis	Chemotherapy and radiation therapy
		Case 2: 5 year 4 months: Female	Chylous exudate, non-malignant	Secondary to respiratory infection and damaged abdominal lymphatics	Antibiotics alone with chemotherapy
		Case 3: 5 years; Female	Bilateral serous, non-malignant	Hypoproteinemia	Spontaneous
4	Kupeli *et al.* (2007) ^6^	10 years; Female	Non-malignant effusion	Secondary to tru-cut biopsy	Spontaneous resolution (although chemotherapy was being given)
5	Al-Hadidi *et al.* (2020) ^7^	12 years; Female	Malignant effusion	Secondary to pleural metastasis	Chemotherapy and lung radiation therapy
6	Canpolat and Jaffe (1995) ^8^	12 years; Female	Malignant effusion	Metastasis	Chemotherapy alone
7	Schinstine *et al.* (2006) ^9^	9 years; Male	Malignant effusion	Secondary to metastasis	Chemotherapy

There is no consensus on the treatment of pleural effusion in WT. Canopolat *et al*. have documented the efficacy of chemotherapy alone in considerably resolving pleural effusion and noted a decrease in tumour size as well. Radiation therapy has also been documented to resolve pleural effusion. ^5^ In our patient, although a therapeutic thoracocentesis was performed to reduce the acute symptoms, the pleural effusion resolved completely by chemotherapy alone. Moreover, CT thorax and pleural fluid cytology did not show evidence of any metastatic disease, hence, radiation therapy to lungs was not implemented.

## Conclusions

Although pleural effusion is a rare occurrence in cases of WT, it need not be synonymous with metastatic disease and can be treated effectively with a good outcome. We recommend a careful strategy in cases presenting with pleural effusion, so as to avoid chemotherapy and radiation therapy-related morbidities. The lack of consensus on management of these groups of patients necessitates further studies in determining risk factors as well as management strategies.

## Patient consent

Written informed consent was obtained from the patient and their parents for their anonymized information to be published in this article.

## Author contributions

PZJ, KV was a major contributor for writing this manuscript and patient care. AD was majorly involved in the chemotherapy management. AA, ARR were overlooking the patient’s management and corrected the final manuscript. JV critically reviewed the abstract section as well as the final manuscript. All the authors have read and approved of the final manuscript.

## Data Availability

All data are available as part of the article and no additional data sources are required.
