# Supplementary material for: Case Report: Pleural effusion in Wilms tumor – always malignant?
Source: F1000Res. 2023 Aug 30;12:1056. [Version 1] doi: 10.12688/f1000research.138794.1 (PMC11263906; doi:10.12688/f1000research.138794.1)
Supplement: Peer review history

# Reviewer report: Reviewer response for version 1

**DOI:** 10.5256/f1000research.152024.r202399

**Published:** 2024-07-22

**Authors:** Keerti Swarnakar

**Affiliations:** 1 Department of Pediatrics, All India Institute of Medical Sciences, Bhopal, Madhya Pradesh, India

Dear Authors, 

   

 Thank you for submitting "Pleural Effusion in Wilms Tumor – Always Malignant? A Case Report." In cases of Wilms tumor (WT) presence of pleural effusion either at the presentation or developing during the course of disease, it’s always important to decide whether it's malignant or non-malignant. In your case report detailed account of the eleven-year-old patient is insightful, highlighting the non-metastatic nature of pleural effusion in Wilms tumor. The comprehensive clinical presentation, effective management with chemotherapy, and thoughtful follow-up plan provide valuable guidance for clinicians. Your comparison with other cases and discussion on the etiology and prognosis enrich the medical literature. This case report is an excellent educational resource, offering practical insights into managing similar presentations. 

 Brief Description of the case-  

 This case report details an eleven-year-old girl with Wilms tumor presenting with a right-sided abdominal mass and massive pleural effusion. After four weeks of chemotherapy, the pleural effusion resolved almost completely. The case highlights that pleural effusion in Wilms tumor patients does not necessarily indicate metastatic disease and can have a favorable prognosis with appropriate treatment. 

 Minor Corrections-  

 1. Kindly add 'Case Report' in the keywords as it is required under the Care Guidelines. 

 2. In the case presentation- Specify the exact duration between the onset of symptoms and the diagnosis. This will provide a clearer understanding of the disease progression and the promptness of the medical intervention. 

 3. In the case presentation - Mention the full form of SpO2 and also its value for better understanding. 

 4. Mention whether Immunohistochemistry and immunofluorescence done or not.  

 5. Please comment whether tuberculosis was ruled out or not.

Are enough details provided of any physical examination and diagnostic tests, treatment given and outcomes?

Yes

Is the case presented with sufficient detail to be useful for other practitioners?

Yes

Is sufficient discussion included of the importance of the findings and their relevance to future understanding of disease processes, diagnosis or treatment?

Yes

Is the background of the case’s history and progression described in sufficient detail?

Yes

Reviewer Expertise:

Pediatrics

I confirm that I have read this submission and believe that I have an appropriate level of expertise to confirm that it is of an acceptable scientific standard.

## References

[1] : Malignant Pleural Effusion: Diagnosis and Management. *Can Respir J* .2020;2020: 10.1155/2020/2950751 2950751 10.1155/2020/2950751 33273991 PMC7695997

[2] : Wilms Tumor with Pleural Metastasis. *Glob Pediatr Health* .2020;7: 10.1177/2333794X20952292 2333794X20952292 10.1177/2333794X20952292 32923526 PMC7457637

# Reviewer report: Reviewer response for version 1

**DOI:** 10.5256/f1000research.152024.r246039

**Published:** 2024-03-07

**Authors:** Marcello Della Corte, Erica Clemente

**Affiliations:** 1 University of Turin, Orbassano, Italy; 2 Medical Sciences - Infectious and Tropical Diseases, University of Turin (Ringgold ID: 9314), Turin, Piedmont, Italy

"Dear Authors,

 Thank you for submitting this manuscript. Your case report offers a valuable overview on Wilms tumor, focusing on an infrequently reported clinical finding: non-malignant pleural effusion. This article highlights that pleural effusion presenting in patients with Wilms tumor needs not to be considered a metastatic disease and can actually have a good prognosis. It represents a new finding in current literature and will certainly be useful in clinical practice.

 I read it with interest and I sincerely cannot find "major points" to be addressed, since your work is already valuable and well structured. I just would add few minor points as suggestions in order to further improve its quality.

 Minor points:

- The introduction is complete, clear and well understandable. I just would suggest to add "case report" to the key words, as stated in CARE Case Report Guidelines ( https://www.care-statement.org/checklist).
- The "Case presentation" section already is really comprehensive, I just have some suggestions in order to further improve its quality.
- "Cinical findings" paragraph:
- Please specify the SPO2 on room air value, I think it is the only missing information in this detailed paragraph.
- "Therapeutic intervention":
- I think the first sentence would be clearer if the Authors replaced the first "her" with "the patient's".
- "Discussion":
- What do the Authors mean by "primary metastasis"? Did they mean "first"?

Are enough details provided of any physical examination and diagnostic tests, treatment given and outcomes?

Yes

Is the case presented with sufficient detail to be useful for other practitioners?

Yes

Is sufficient discussion included of the importance of the findings and their relevance to future understanding of disease processes, diagnosis or treatment?

Yes

Is the background of the case’s history and progression described in sufficient detail?

Yes

Reviewer Expertise:

Urology, Uro-oncology, Andrology, Pediatric Urology

We confirm that we have read this submission and believe that we have an appropriate level of expertise to confirm that it is of an acceptable scientific standard.

# Response

**Published:** 2024-03-07

**Authors:** Keta Vagha

**Affiliations:** Pediatrics, Jawaharlal Nehru Medical College, Sawangi (Meghe), Wardha, Maharashtra, India

I am extremely grateful for your positive comments. I shall make the corrections advised by you and resubmit the manuscript.
